# Trends in the types and quality of childhood immunisations research output from Africa 1970–2010: mapping the evidence base

**DOI:** 10.1186/1472-6963-14-52

**Published:** 2014-02-04

**Authors:** Shingai Machingaidze, Gregory D Hussey, Charles S Wiysonge

**Affiliations:** 1 Vaccines for Africa Initiative (VACFA), Institute of Infectious Diseases and Molecular Medicine (IDM), University of Cape Town, Cape Town, South Africa; 2 Division of Medical Microbiology, Department of Clinical Laboratory Sciences, University of Cape Town, Cape Town, South Africa; 3 Centre for Evidence-based Health Care, Stellenbosch University, Tygerberg, Cape Town, South Africa; 4 Division of Community Health, Department of Interdisciplinary Health Sciences, Faculty of Medicine and Health Sciences, Stellenbosch University, Tygerberg, Cape Town, South Africa

**Keywords:** Expanded Programme on Immunisations (EPI), Immunisations programmes, Childhood immunisations, Vaccines, Children, Africa

## Abstract

**Background:**

Over the past four decades, extraordinary progress has been made in establishing and improving childhood immunization programmes around Africa. In order to ensure effective and sustainable positive growth of these childhood immunisations programmes, the development, adaptation and implementation of all interventions (programme activities, new vaccines, new strategies and policies) should be informed by the best available local evidence.

**Methods:**

An assessment of the peer-reviewed literature on childhood immunization research published in English from 1970 to 2010 was conducted in PubMed and Africa-Wide databases. All study types were eligible for inclusion. A standard form was used to extract information from all studies identified as relevant and entered into a Microsoft Access database for analysis.

**Results:**

Our initial search yielded 5436 articles from the two databases, from which 848 full text articles were identified as relevant. Among studies classified as clinical research (417), 40% were clinical trials, 24% were burden of disease/epidemiology and 36% were other clinical studies. Among studies classified as operational research (431), 77% related to programme management, 18% were policy related and 5% were related to vaccine financing. Studies were conducted in 48 African countries with six countries (South Africa, The Gambia, Nigeria, Senegal, Guinea-Bissau and Kenya) accounting for 56% of the total research output. Studies were published in 152 different journals with impact factors ranging from 0.192 to 53.29; with a median impact factor of 3.572.

**Conclusion:**

A similar proportion of clinical versus operational research output was found. However, an uneven distribution across Africa was observed with only six countries accounting for over half of the research output. The research conducted was of moderate to high quality, with 62% being published in journals with 2010 impact factors greater than two. Urgent attention should be given to the development of research capacity in low performing countries around Africa, with increased focus on the process of turning immunisations programme research evidence into policy and practice, as well as increased focus on issues relating to vaccine financing and sustainability in Africa.

## Background

Immunisations is one of the most cost-effective public health interventions that have resulted in significant reductions in global child morbidity and mortality [1]. The focus of immunisations in low- and middle-income countries (LMICs) has been the Expanded Programme on Immunisations (EPI), which was launched in 1974 by the World Health Organisation (WHO) following the successful programme for the eradication of smallpox [2]. The programme consisting of scheduled vaccination visits free of charge for children less than one year of age is operated by the Ministries of Health in respective countries and supported in some cases by international agencies such as WHO, the United Nations Children Fund (UNICEF) and the Global Alliance for Vaccines and Immunisations (GAVI), as well as various other donor agencies. National EPI’s function as a series of inter-related components (i.e. vaccine supply and quality, surveillance, management, communication and advocacy, logistics and capacity building) that aim to improve immunisations coverage and reduce child morbidity and mortality [3].

While combined global, regional, and national efforts over the past four decades to improve EPI have resulted in the global coverage with the third dose of diphtheria-tetanus-pertussis vaccine by 12 months of age (DTP3) reaching 85% in 2010, the WHO African Region reached only 77% in 2010 [4-8]. As Africa continues to address programmatic and policy issues related to improving childhood immunisations, it is necessary for the development, adaptation and implementation of all interventions (programme activities, new vaccines and new strategies) to continue to be informed by the best available local evidence [9-12]. As such, the increase in childhood immunisations coverage in Africa over the years should have been accompanied by similar growth in available childhood immunisations literature. A recent bibliometric analysis assessed factors associated with childhood immunisations research productivity in Africa and found no association between research productivity and immunisations coverage. In addition, the study found immunisations research productivity in Africa to be highly skewed, with private health expenditure (possibly reflecting country economic status) having a statistically significant positive association with immunisations research productivity [3]. Africa’s contribution to the global research on childhood immunisations has been minimal [3].

We recognise that emphasising the general need for increased research on childhood immunisations in Africa without assessing and identifying exactly what areas of research are lacking would be counter-productive. As a first step to improving this situation, we need detailed information on the type of research that is being conducted in Africa.

The objective of this study was to map the evidence base of published literature on childhood immunisations research in Africa published in English from 1970 to 2010 in order to ascertain the types and quality of childhood immunisations research output on the continent.

## Methods

### Search strategy

We searched two literature databases for relevant peer-reviewed articles published from January 1, 1970 to December 31, 2010. These were PubMed and Africa-Wide databases (*Africa-Wide Information-via EBSCO host combines databases {African Studies, South African Studies, and African Healthline} to form a multidisciplinary aggregation offering unique and extensive coverage of all facets of Africa and African studies. This resource is essential for those with an interest in African research, and information on and about Africa*[13]). The development and implementation of the search strategy was conducted by the first author [SM] in consultation with the other authors [CSW and GH]. We used a combination of MESH (Medical Sub-Headings) terms in PubMed and key word search terms in Africa-Wide. The following search phrases were used and combined using the Boolean operator “AND”: (1) ‘immunisations’ or ‘vaccination’ or ‘immunisations programmes’ (and several derivatives including ‘immunis’ , ‘immuniz’ , ‘immunisations schedule’ , ‘vaccine’ , ‘vaccinate’ , ‘mass vaccination’); (2) the names of each individual African country were included in its various forms (for example Mozambique and Mocambique for Mozambique); and (3) ‘infant’ or ‘newborn’ or ‘child’ or ‘children’. For the latter, MESH terms such as “Infant”, “Newborn” [MESH], “Child, Preschool” [MESH] were also used in PubMed. Reference lists of selected articles were searched to identify additional relevant papers. Only published studies, written in English were included in this review (due to limited resources as well as the large outputs obtained from the databases selected). Studies not conducted in humans were excluded from further analysis. Duplicate articles obtained in both databases were noted and only one was included in the final analysis.

The titles and abstracts obtained from the initial search were reviewed by one author [SM] and verified by a second author [CSW]. Articles were considered relevant if they (1) were about immunisations/vaccines or immunisations programmes and policies; (2) were studies conducted in Africa; and (3) were studies in children. Articles reporting findings from studies of adult vaccination only, studies not conducted in African populations or studies merely reporting the epidemiology of vaccine preventable diseases without specific reference to vaccination programmes were excluded. A total of 53 African countries were considered for this analysis (regardless of classification by WHO into African or Eastern Mediterranean Regions).

### Article review and analysis

The full text of relevant publications was obtained online (if available), from the University of Cape Town’s Health Science Library, or from the inter-library loan request service of the Health Science Library. If the full text was not obtained via these sources, the article was classified as ‘not found’. Full text articles were reviewed by one author [SM], and data from selected articles were abstracted using a standardised data extraction form designed in consultation with all authors. Abstracted information was then entered into a Microsoft Access database designed and managed by SM. All study types were eligible for inclusion (descriptive, qualitative and intervention studies). Studies were classified as either ‘Clinical Research’ (clinical trials phase one to four; burden of disease/epidemiology; other clinical studies) or ‘Operational Research’ (programme management; policy; vaccine financing). Once entered into the database and classified accordingly by one author, a random sample of 100 articles from the database was assessed by all authors to ensure correct classification of studies.

### Quality of research

A study published in a journal with an impact factor of greater than zero but less than two was said to be of ‘moderate quality’; a journal with an impact factor of two to five was said to be of ‘good’ quality; a journal with an impact factor of five to ten of ‘very good’ quality; and any journal with an impact factor greater than ten of ‘excellent’ quality. This classification was produced by the authors as a proxy for measuring the quality of research.

## Results

**Figure 1:**
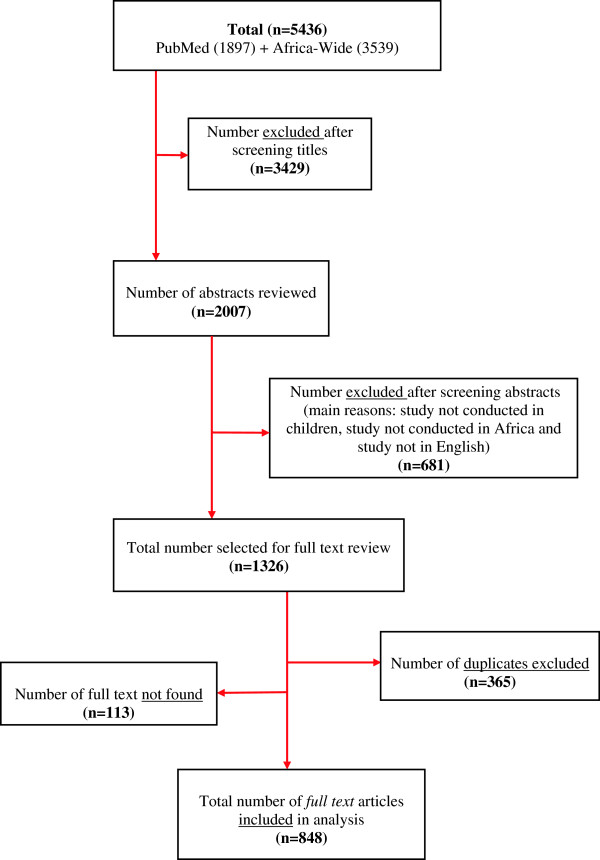
Study selection process.

Our searches yielded 5436 articles; 1897 articles from PubMed and 3539 articles from Africa-Wide (Figure 1). Initial review of titles from both databases identified 2007 articles as potentially relevant while 3429 were deemed not relevant and excluded. After review of the 2007 abstracts, a further 681 articles were excluded. The main reasons for exclusion at this stage were: study not conducted in Africa and study not published in English. A total of 1326 articles were identified as relevant from the two databases. From these, 365 articles were identified as duplicates. The full text of 113 articles were ‘not found’, resulting in a total of 848 full text articles being included in the analysis presented in this study.

### Distribution of studies

A progressive increase in the total volume of childhood immunisations research output in Africa was observed with each successive decade: 55/848 (6 · 5%) from 1970 to 1980; through 178/848 (21%) from 1981 to 1990 and 284/848 (33 · 5%) from 1991 to 2000; to 331/848 (39%) from 2001 to 2010. The last two decades were found to be the most productive with 615/848 (72%) of the research output analysed here being published between 1991 and 2010.

**Figure 2:**
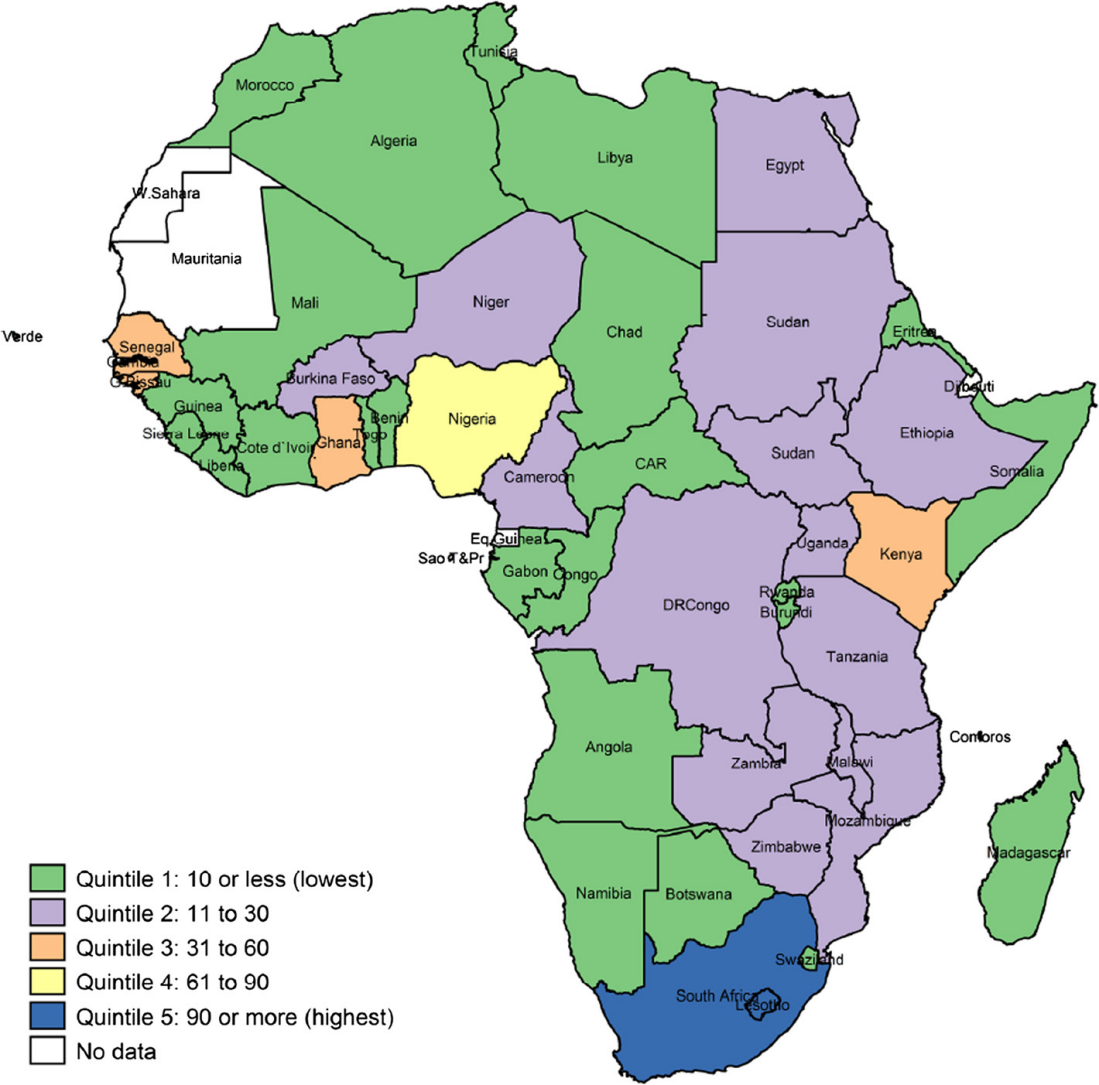
Distribution of childhood immunisations publication across Africa.

Included studies were conducted in 48 African countries (Figure 2). The six top ranking countries with regards to childhood immunisations research studies included in this analysis were: South Africa (n = 182, 21%), The Gambia (n = 89, 10%), Nigeria (n = 84, 9%), Senegal (n = 53, 6%), Guinea-Bissau (n = 48, 5%), and Kenya (n = 44, 5%). These six countries combined accounted for 56% of the total research output included in this analysis.

### Classification of studies

A similar proportion of clinical versus operational research output was found. A total of 417/848 (49%) articles were classified as clinical research. Of these, 168 (40%) were clinical trials (phase one to phase four); 149 (36%) were other clinical studies (largely studies assessing immune response to various licensed vaccines or adverse events following immunisations); and 100 (24%) were burden of disease or epidemiology studies on diseases targeted by traditional and newly introduced EPI vaccines. A total of 431/848 (51%) articles were classified as operational research. Of these, 332 (77%) related to vaccine programme management (largely studies assessing vaccine coverage, service delivery and logistics, management, as well as communication and advocacy); 78 (18%) were policy related; and 21 (5%) were related to vaccine financing. This classification of studies is shown in Table 1.

**Table 1:** Classification of selected studies

**Categories**	**Number of studies (%)**
**Clinical research**	**417**
Clinical trials (Phases1-4)	168 (40%)
Burden or disease/epidemiology	100 (24%)
Other clinical studies	149 (36%)
**Operational research**	**431**
Programme management	332 (77%)
Policy	78 (18%)
Vaccine financing	21 (5%)

Our analysis shows that over the past four decades measles was the leading vaccine-preventable disease studied by researchers in and around Africa. Of the 848 articles, half (426) were studies on four diseases: 206 (24%) were on measles, 77 (9%) on poliomyelitis, 75 (8 · 8%) on tuberculosis [bacille Calmette-Guérin (BCG)] and 68 (8%) on hepatitis B. Of the remaining half, 135 (16%) were studies on nine other vaccine-preventable diseases namely: 31 (3 · 7%) on meningococcal disease, 27 (3 · 2%) on *Haemophilus influenzae* type b (Hib), 24 (2 · 8%) on pneumococcal disease, 16 (1 · 9%) on neonatal tetanus, 14 (1 · 7%) on pertussis, 10 (1 · 2%) on diarrhoea and 12 (1 · 4%) on yellow fever, diphtheria and smallpox. A small proportion of studies (n = 27, 3 · 2%) were on integration of EPI to provide other primary care services, and 260 (31%) targeted multiple vaccine-preventable diseases with more than one vaccine given in each study.

### Quality of research

**Figure 3:**
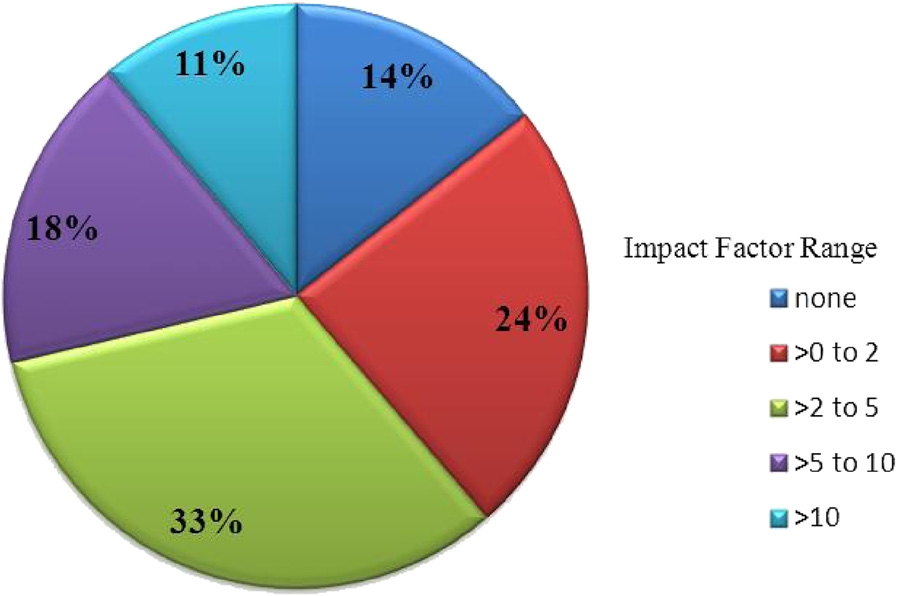
Proportion of publications with varying impact factors.

Studies were published in 152 different journals. The impact factors of the journals ranged from 0.192 to 53.29. The median impact factor was found to be 3.572. The 2010 impact factor for 41/152 (27%) journals was not found or the journal did not have an impact factor; 37/152 (24%) had an impact factor greater than zero to two; 51/152 (34%) from greater than two to five; 14/152 (9%) from greater than five to ten; and 9/152 (6%) had an impact factors greater than ten. The journals, total number of publications from that journal, as well as the 2010 impact factors for the 15 journals with the highest number of publications are listed in Table 2. The South African Medical Journal was found to have the highest number of articles (101 articles; 2010 journal impact factor 1.676), followed by Vaccine (82 articles; 2010 impact factor 3.572), The Lancet (69 articles; 2010 impact factor 33.633), Journal of Infectious Diseases (51 articles; 2010 impact factor 6.288), Bulletin of the World Health Organisation (41 articles; 2010 impact factor 5.459) and the Pediatric Infectious Diseases Journal (34 articles; 2010 impact factor 3.064). Figure 3 shows the distribution of publications in different journals with varying impact factors.

**Table 2:** Top 15 journals and their impact factors with African researchers as first authors

**Journal**	TP*	2010 IF*
*South African Medical Journal*	101	1.676
*Vaccine*	82	3.766
*The Lancet*	69	33.633
*Journal of Infectious Diseases*	51	6.288
*Bulletin of the World Health Organization*	41	5.459
*Pediatric Infectious Diseases Journal*	34	3.577
*Royal Society of Tropical Medicine and Hygiene*	29	2.615
*Journal of Tropical Pediatrics*	28	1.388
*East African Medical Journal*	28	n/a
*International Journal of Epidemiology*	25	6.414
*Tropical Medicine and International Health*	18	2.795
*Annals of Tropical Paediatrics*	18	0.9
*Social Science & Medicine*	13	2.699
*Central African Journal of Medicine*	12	n/a
*British Medical Journal*	11	13.471

*TP = Total Publications out of 848; IF = Impact Factor; *n/a - 2010 impact factor was not found online or the journal did not have an impact factor.

## Discussion

This study identified several trends regarding the types and quality of childhood immunisations research output in Africa over the past four decades. An important finding from this study was the even distribution between clinical versus operational research. Childhood immunisations programmes are a combination of several different components which in turn means that efforts to address programmatic and policy issues related to the improvement of these programmes must continue to be informed by both clinical and operational evidence. The development of an efficacious and effective vaccine (clinical research) without an adequate national immunisations programme to ensure prompt and safe delivery of the vaccine to all children (operational research) is suboptimal and will result in under-vaccinated and unvaccinated children.

An important component of health systems strengthening regarding childhood immunisations programmes is the efficient translation of research findings into policy and practice. Only a small proportion (18%) of studies classified as operational research addressed policy issues on childhood immunisations, with the majority of the articles having been written by authors with South African affiliation and published in the South African Medical Journal. African countries need to pay urgent attention to ensuring that sufficient policy-related research is conducted to ensure that individual country childhood immunisations policies are informed by current and relevant research evidence.

Another important aspect with regards to immunisations systems strengthening in Africa is that of vaccine/immunisations financing. In a recent report, Médecins Sans Frontieres (MSF) highlights that vaccine procurement and pricing strategies, as well as vaccine adaptation to suit low and middle income countries (LMICs) remain essential components of helping to strengthen immunisations systems across Africa [14]. With the cost per live birth for immunisations in LMICs expected to increase from US$25 for traditional EPI vaccines (including hepatitis B and Hib vaccines) to US$58 if pneumococcal conjugate vaccine (PCV) and rotavirus vaccine are added to the schedule [2], vaccine financing should remain high on the childhood immunisations research agenda in Africa. Only 5% of studies classified as operational research addressed vaccine financing in Africa, suggesting that there is room for much improvement in this area.

Similar to the findings of a bibliometric analysis [3] of childhood immunisations research productivity in Africa from 1974 to 2010, we found that the distribution of childhood immunisations research across Africa was highly skewed with six countries (South Africa, The Gambia, Nigeria, Senegal, Guinea-Bissau and Kenya) accounting for slightly over half of the total research output analysed. This highly uneven distribution across Africa is a reflection of the limited and under-developed research capacity in most African countries. Multiple medical schools, public health schools as well as local and international health research institutions present in the above mentioned six countries are a likely explanation of the high volumes of childhood immunisations research when compared to other African countries. However, it is important to note that although it is beyond the scope of this paper, the individual country economic development status (amongst other individual country characteristics) is likely to influence research productivity as some countries have far more resources and increased capacity to both conduct research and implement findings when compared to others [3,15,16].

We identified measles as the leading vaccine-preventable disease targeted by African researchers over the past four decades and this is not surprising as it was the leading cause of infant and child mortality in Africa for many years. Greater research attention was also given to poliomyelitis, tuberculosis [bacille Calmette-Guérin (BCG)] and hepatitis B when compared to other vaccine-preventable diseases. Childhood immunisations research focus is likely to have shifted and will shift in future according to burden of disease as well as child mortality patterns in Africa. In a recent publication on causes of global child mortality as of 2010, pneumonia was identified as the leading cause of death contributing 18% all-cause mortality in children under 5 years compared to measles (1%), diarrhoea (11%) and malaria (7%) [17]. The volume of research in Africa on childhood immunisations in relation to pneumonia (PCV vaccine, Hib vaccine) and diarrhoea (rotavirus vaccine), amongst other diseases, has increased in recent years and will continue to do so as current epidemiological patterns demand attention be shifted to these leading child killer diseases [18,19].

EPI in Africa may very well be the most successful health programme on the continent, and is thus ideal for providing add-on services for improving health status of young children and mothers [6,20,21]. A small proportion of studies included in this analysis (3.2%) related to the integration of other primary health care services to EPI. This integration of services such as the distribution of insecticide-treated bed nets, vitamin A supplementation, and deworming of children, is likely to improve as immunisations programmes around Africa become stronger and better able to facilitate additional interventions besides scheduled vaccines. However, accomplishing this expanded agenda will require a firm commitment from African governments both politically and financially [22]; and will also require governments and their development partners to establish efficient mechanisms to facilitate this expansion in their respective primary health care systems [20-23].

The quality of research is difficult to measure. Impact factors provide a measure of the frequency peer-reviewed journal articles are cited by other researchers as well as a yearly measure of the comparative importance of a journal in relation to other journals in its field. We used impact factors as a proxy measure of the quality of childhood immunisations research in Africa, where studies published in journals with higher impact factors were said to be of higher quality. In general, the majority of articles were of ‘good’ quality (i.e. impact factor of two to five), while smaller proportions were of ‘very good’ and ‘excellent’ quality (i.e. impact factors of five and above).

There were several limitations to the analysis presented here. We acknowledge that by searching only two databases we may have missed out on relevant articles indexed in other databases. In addition, not all local country journals are indexed in PubMed and Africa-Wide, meaning studies published in these non-indexed journals may have been missed. An assessment of the grey literature with regards to immunisations programmes was not included as part of this review. This means that relevant operational and policy-related documents in the grey literature were not included in this analysis [24-26]. Due to limited resources, studies published in languages other than English were excluded. In addition, due to the broad nature of childhood immunisation (encompassing many different components) identifying a search strategy that includes all aspects of childhood immunisations was challenging. Quality of research was assessed using journal impact factors classified by the authors as a proxy, and this may not be an accurate reflection of the true quality of the individual studies. Despite these limitations, we believe that this analysis is still sufficient to provide a representative and much needed evidence base of the types and quality of childhood immunisations research in Africa over the past four decades.

African countries and international agencies assisting Africa should prioritise operational research in the context of GAVI and the Decade of Vaccines/Global Vaccine Action Plan (GVAP). The mission of the GVAP is to extend, by 2020 and beyond, the full benefits of immunisations to all people, regardless of where they are born, who they are or where they live. The plan calls for creation of a robust research and development environment for immunisations, and urges academia to “engage more with systematic reviews to identify areas where solid scientific evidence exists (which should be the basis of health policies) and those areas where such evidence is lacking (which would be the basis for future primary research)”. Examples of systematic reviews could include understanding interventions for improving routine immunisations coverage, the effects of mass vaccination campaigns on routine immunisations services, financial arrangements for immunisations and other primary healthcare programmes, and the effectiveness of interventions for expanding routine immunisations services beyond childhood. Beyond systematic reviews of global evidence, there is need for local evidence on perceptions, needs, values and preferences of immunisations stakeholders as well as socio-cultural and religious factors affecting immunisations in African communities.

## Conclusion

While a progressive increase in the volume of childhood immunisations research output was observed with each successive decade since 1970, the last two decades (1991–2010) were found to be the most productive. An even distribution between clinical versus operational research was found with regards to childhood immunisations research output in Africa. However, the distribution of studies across Africa was found to be highly skewed, with six countries (South Africa, The Gambia, Nigeria, Senegal, Guinea-Bissau and Kenya) accounting for half of the total research output. Measles was found to be the leading vaccine-preventable disease studied by African authors over the past four decades. Research was of moderate to excellent quality, with 62% being published in journals with impact factors greater than two. Urgent attention should be given to the development of research capacity in low performing countries around Africa, with increased focus on the process of turning immunisations programme research evidence into policy and practice, as well as increased focus on issues relating to vaccine financing and sustainability in Africa.

## Competing interests

The authors declare that they have no competing interests.

## Authors’ contributions

SM contributed towards the study conception and design, study screening and selection, data extraction, wrote the first and subsequent drafts of the manuscript, and finalised submission of the manuscript. GH contributed towards study design, study selection, and reviewed all versions of the manuscript. CW contributed towards study design and conception, study screening and selection, and reviewed all versions of the manuscript. All authors read and approved the final manuscript.

## Pre-publication history

The pre-publication history for this paper can be accessed here:

http://www.biomedcentral.com/1472-6963/14/52/prepub
